# Evaluating the Published Critical Care Research from the World Health Organization Eastern Mediterranean Region

**DOI:** 10.1186/s13104-019-4093-7

**Published:** 2019-01-18

**Authors:** Mohamad Elaibaid, Lama H. Nazer, Lama Shaikha, Nada Al-Qadheeb, Ruth Kleinpell, Keith M. Olsen, Feras Hawari

**Affiliations:** 1 Department of Medicine, Ibn Sina University, Khartoum, Sudan; 2 Department of Pharmacy, King Hussein Cancer Center, Queen Rania Al-Abdallah Street, PO Box 1269, Amman, 11941 Jordan; 3 Department of Medicine, Hafer Albatin Central Hospital, Hafer Albatin, Saudi Arabia; 4 Vanderbilt University School of Nursing, Nashville, TN USA; 5 College of Pharmacy, University of Nebraska, Omaha, NE USA; 6 Section of Pulmonary and Critical Care, King Hussein Cancer Center, Amman, Jordan

**Keywords:** Critical care, Research, Research productivity, Research output, Eastern Mediterranean, Developing countries

## Abstract

**Objectives:**

Evaluation of published research in a region provides insight into relevant aspects of clinical care and research priorities. This study aimed to provide a comprehensive assessment of the type of critical care research published in the World Health Organization Eastern Mediterranean region (EMR) over a 10-year period.

**Results:**

During the study period (2007–2016), the search strategy revealed 4303 publications, of which 1537 were included in the analysis; studies were excluded for the following reasons: not critical care, conducted in non-EMR countries, editorials, case reports, in-vitro or animal studies, as well as those conducted in multiple countries and those that evaluated foreign military personal. Countries varied in the number of publications produced, ranging from none in Somalia to 620 in Iran. The majority of the studies were observational (78%), evaluated adults (73%), and the most common areas of research were infectious (29%) and respiratory (10%) diseases. Median sample size was 120 and the mean (SD) impact factor of the journals in which the articles were published was 1.02 (0.7).

## Introduction

Critical care research forms the foundation for evidence-based practice [1, 2]. While the Eastern Mediterranean Region (EMR) has made great advances in the field of medicine, including critical care, the proportion of resources allocated to research is generally low [3]. The EMR of the World Health Organization (WHO) comprises 22 countries [4]. The healthcare services in these countries vary widely; for example, the number of hospital beds per 1000 population ranges from 0.5 in Afghanistan to 3.7 in Libya [5].

Bibliometric studies of research in various medical disciplines in the EMR demonstrate overall low research productivity with considerable variation among countries [6–12]. Furthermore, the quality of research and the impact factor of journals in which it is published is generally low [10, 13].

Two studies examined research productivity in critical care globally but did not report on research in the EMR [14, 15]. Recently, Nazer et al. [16] published a brief report providing a quantitative assessment of published critical care research in the EMR. Though the authors reported a substantial increase in research output in the last decade, it was generally low and varied among countries. This study extended the work and aimed to provide more insight about the current state of critical care research in the EMR countries through a comprehensive assessment of the type of critical care research published over a 10-year period.

## Main text

### Methods

#### Search strategy

Two investigators (ME and LN) independently conducted a PubMed search to identify original articles and reviews in the field of critical care conducted in countries in the EMR (January 1, 2007–December 31, 2016). The search terms used were “critical care”, “critical illness”, “intensive care”, “intensive care unit”, “critical care outcome”, and “critically ill”. The search was performed for publications from each of the EMR countries by combining the search terms as medical subject headings (MeSH) and as text words in all fields with the name of the country as MeSH term and as text word, with no language restrictions. A medical librarian reviewed the search strategy. The results of each investigator were compared, and any discrepancies were discussed. If a decision could not be reached, the discrepancy was presented to all study investigators, and a final decision was made by majority consensus.

#### Trial selection and eligibility

Eligible articles were those that included patients from ICUs in the EMR. Studies of critically ill foreign military personnel and military medical practice in EMR countries were excluded, as it was considered that those would reflect the Western clinical practice rather than that of the country in which the study was conducted. Studies of critically ill patients in non-ICU settings were included if the main objective of the study was to investigate a critical illness. Meta-analyses, reviews, and clinical practice guidelines were included if the primary author was from one of the EMR countries. Studies conducted in more than one country were excluded since those may include non-EMR countries or may not have originated from the EMR and therefore, would not be a reflection of research productivity of the countries involved. However, multicenter studies in which all study sites were in the same country were included in the review. Publications on anesthesia or intraoperative management, editorials, letters to the editor, case reports, in-vitro investigations, animal studies, and conference proceedings were also excluded.

The total number of publications from each country was determined, as well as the number of publications adjusted for the population and the number of publications adjusted for the gross domestic product (GDP). The most recent data reported by the World Bank was used to determine the population and GDP for each country [5].

For clinical studies, the location in which the study was conducted was the country of origin, regardless of the location of the primary author. However, for meta-analysis, reviews, and clinical practice guidelines, the country of origin was that for the primary author.

A comprehensive assessment was conducted for critical care research published by assessing the patient population, research field, impact factor (IF), H-index, sample size, and study design for each eligible publication.

Patient populations were classified into: adults, pregnant women, pediatrics, and neonates. The research field was categorized for each publication according to the classification described in Table 2. For publications that addressed more than one research field, the major field was recorded.

For each publication included in this analysis, the IF of the journal in which it was published was recorded as the most recently reported for the journal at the time of the analysis, regardless of the article’s publication date. The most recent IF was derived from the website for each journal or, if the journal’s website did not provide the information, from the Web of Science [17]. For journals with no reported IF, an IF of zero was recorded.

The H-index reported for each country in the category of critical care and intensive care medicine was recorded. The SCImago Journal and Country Rank database, which is a publicly available portal that lists the journals and country scientific indicators retrieved from information in the Scopus database, were used to identify the H-index for each country [18].

The sample size was taken as the total number of patients, events, or measurements evaluated in the clinical study or meta-analysis. In the sample size analysis, we excluded reviews and studies that did not report their sample size.

The design of all studies during the past decade was classified as: randomized-controlled trial, non-randomized controlled trial, observational study, meta-analysis, or review. The observational studies included retrospective and prospective cohort, case–control, and cross-sectional studies, as well as surveys. Both systematic and non-systematic reviews were included.

### Results

**Fig. 1:**
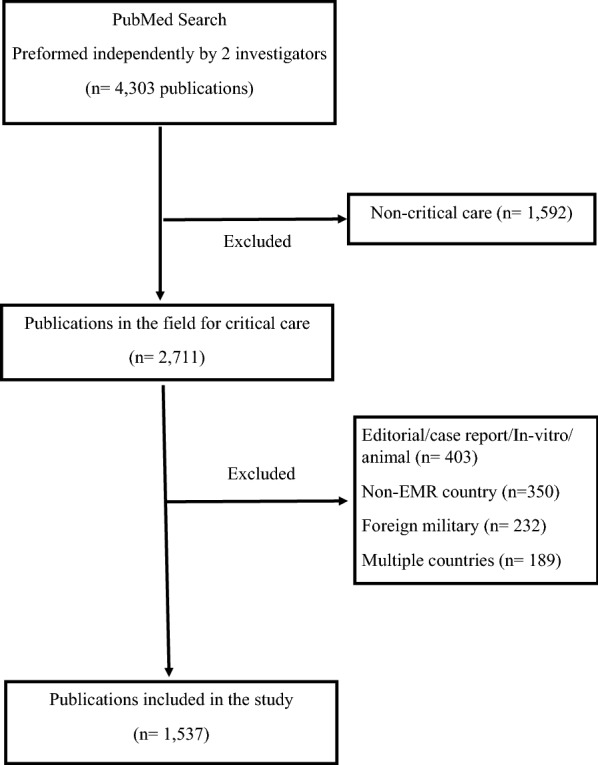
Flow chart of the search strategy to identify published critical care research from the Eastern Mediterranean Region

The search strategy revealed 4303 publications, of which 1537 were included in this analysis; 2766 were excluded for the reasons outlined in Fig. 1. The number of publications produced in the EMR countries varied significantly, ranging from none from Somalia to 620 publications from Iran. Tables 1 and 2 outline the assessment of the published critical research from the EMR countries. The largest number of publications was from Iran (n = 620), Saudi Arabia (n = 273), and Egypt (n = 165). However, when adjusted for the population, the highest number of publications was from Qatar, Tunisia, and Saudi Arabia and when adjusted for GDP, the highest number of publications was from Tunisia, Jordan, and Iran.

**Table 1:** Number of published critical care research from the Eastern Mediterranean Region and the type of patient populations studied

Country	Number of publications			Patient population			
	Total number	Adjusted for population^a^	Adjusted for GDP^b^	Adults N (%)	Children N (%)	Neonates N (%)	Pregnant N (%)
Afghanistan	1	0.028	0.05	1 (100%)	0	0	0
Bahrain	11	7.33	0.31	7 (63.6%)	0	3 (27.3%)	1 (9.1%)
Djibouti	2	2.0	1.1	2 (100%)	0	0	0
Egypt	165	1.70	0.7	82 (49.7%)	21 (12.7%)	59 (35.8%)	3 (1.8%)
Iran	620	7.64	1.4	487 (78.5%)	21 (3.4%)	111 (17.9%)	1 (0.16%)
Iraq	8	0.20	0.04	4 (50%)	0	2 (25%)	2 (25%)
Jordan	63	6.50	1.6	47 (74.6%)	7 (11.1%)	8 (12.7%)	1 (1.6%)
Kuwait	26	6.3	0.2	16 (61.5%)	3 (11.5%)	7 (26.9%)	0
Lebanon	42	6.88	0.81	38 (90.5%)	1 (2.4%)	2 (4.8%)	1 (2.4%)
Libya	5	0.78	0.1	3 (60%)	1 (20%)	1 (20%)	0
Morocco	39	1.09	0.36	26 (66.7%)	6 (15.4%)	2 (5.1%)	5 (12.8%)
Oman	16	3.48	0.22	9 (56.3%)	2 (12.5%)	4 (25%)	1 (6.25%)
Pakistan	95	0.48	0.3	52 (54.7%)	27 (28.4%)	14 (14.7%)	2 (2.1%)
Palestine^c^	5	1.06	0.34	3 (60%)	0	2 (40%)	0
Qatar	36	13.8	0.21	32 (88.9%)	2 (5.6%)	2 (5.6%)	0
Saudi Arabia	273	8.30	0.40	218 (79.8%)	24 (8.8%)	30 (11%)	1 (0.4%)
Sudan	6	0.15	0.05	0	0	5 (83.3%)	1 (16.7%)
Somalia	0	0	0	0	0	0	0
Syria	3	0.16	0.07	3 (100%)	0	0	0
Tunisia	102	8.87	2.5	75 (73.5%)	13 (12.7%)	11 (10.8%)	3 (2.9%)
United Arab Emirates	16	1.7	0.04	11 (68.8%)	1 (6.25%)	4 (25%)	0
Yemen	3	0.11	0.16	3 (100%)	0	0	0

^a^Data reflect the most recent statistics reported by the World Bank, which was from 2017 for all countries as follows in million: Afghanistan 35.5, Bahrain 1.5, Djibouti 0.96, Egypt 97.5, Iran 81.2, Iraq 38.3, Jordan 9.7, Kuwait 4.1, Lebanon 6.1, Libya 6.4, Morocco 35.7, Oman 4.6, Pakistan 197, Palestine 4.7, Qatar 2.6, Saudi Arabia 32.9, Sudan 40.5, Somalia 14.7, Syria 18.3, Tunisia 11.5, United Arab Emirates 9.4, and Yemen 28.3

^b^ Data reflect the most recent statistics reported by the World Bank, which was 2017 for all countries except Yemen (2016) and Syria (2007), as follows in billion US dollar: Afghanistan 20.8, Bahrain 35.3, Djibouti 1.8, Egypt 235.3, Iran 439.5, Iraq 197.7, Jordan 40, Kuwait 120, Lebanon 51.8, Libya 51, Morocco 109.1, Oman 72.6, Pakistan 305, Palestine 14.5, Qatar 167.6, Saudi Arabia 683.8, Sudan 117.5, Somalia 7.4, Syria 40.4, Tunisia 40.2, United Arab Emirates 382.6, Yemen 18.2

^c^ Listed as West Bank and Gaza by the World Bank

**Table 2:** Quality of critical care publications from the Eastern Mediterranean Region reflected by the sample size, study design, research fields, and journal assessment

Country	No.	Sample size median (range)	Study design					Most common research fields^a^			Journal Assessment		
			RCT N (%)	NRCT N (%)	Observational N (%)	Meta-analysis N(%)	Review N(%)	Research field N (%)	Research field N (%)	Research field N (%)	IF Mean (SD)	Journals with IF>3 (no)	H Index
Afghanistan	1	410	0	0	1 (100%)	0	0	ID 1 (100%)	N/A	N/A	0.37	0	6
Bahrain	11	100 (4–1579)	0	0	9 (81.8%)	0	2 (18.2%)	ID 3 (27.3%)	Q/S 3 (27.3%)	Resp 2 (18.2%)	1.31 (1.7)	1	3
Djibouti	2	131 (18–244)	0	0	2 (100%)	0	0	Epidem 1 (50%)	Heme 1 (50%)	N/A	0	0	0
Egypt	165	75 (15–7393)	19 (11.5%)	0	140 (84.8%)	0	6 (3.6%)	ID 68 (41.2%)	Resp 24 (14.5%)	Renal 11 (6.7%)	1.34 (1.3)	16	28
Iran	620	84 (4–18,682)	129 (20.8%)	27 (4.4%)	439 (70.8%)	3 (0.48%)	22 (3.5%)	ID 132 (21.3%)	Resp 68 (11%)	Cardio 40 (6.5%)	0.54 (1.9)	13	33
Iraq	8	222 (28–986)	0	0	6 (75%)	0	2 (25%)	ID 2 (25%)	Q/S 2 (25%)	Burns 2 (25%)	1.32 (0.7)	0	10
Jordan	63	135 (6–10,792)	1 (1.6%)	1 (1.6%)	58 (92.1%)	0	3 (4.8%)	ID 14 (22.2%)	Epid 7 (11.1%)	Resp 6 (9.5%)	1.61 (1.6)	6	11
Kuwait	26	94 (5–10,419)	0	0	24 (92.3%)	0	2 (7.7%)	ID 15 (57.7%)	Epid 3 (11.5%)	Q/S 2 (7.7%)	1.63 (1.0)	2	17
Lebanon	42	66 (10–1506)	0	3 (7.1%)	32 (76.2%)	0	7 (16.7%)	ID 10 (23.8%)	Resp 6 (14.3%)	Burn 5 (12%)	2.00 (2.0)	5	21
Libya	5	423 (260–995)	0	0	5 (100%)	0	0	ID 2 (40%)	Q/S 2 (40%)	Cardio 1 (20%)	0.40 (0.4)	0	1
Morocco	39	145 (9–146)	2 (5.1%)	1 (2.6%)	36 (92.3%)	0	0	ID 9 (23%)	Resp 4 (10.3%)	Q/S 4 (10.3%)	1.9 (2.9)	5	11
Oman	16	65 (29–95)	0	0	15 (93.8%)	0	1 (6.3%)	ID 3 (18.8%)	Resp 2 (12.5%)	Epid 2 (12.5%)	0.58 (1.1)	0	10
Pakistan	95	144 (3–5800)	2 (2.1%)	0	89 (93.7%)	0	4 (4.2%)	ID 33 (34.7%)	Epid 10 (10.5%)	CV 9 (9.5%)	1.0 (1.2)	5	17
Palestine	5	123 (13–275)	0	0	5 (100%)	0	0	P/F 2 (40%)	ID 2 (40%)	CPR 1 (20%)	1.13 (1.6)	1	2
Qatar	36	201 (36–1036)	1 (2.8%)	0	33 (91.7%)	0	2 (5.6%)	ID 4(11%)	Epid 4 (11%)	Org Don 4 (11%)	1.35 (1.1)	1	11
Saudi Arabia	273	130 (6–256,195)	11 (4%)	2 (0.73%)	214 (78.4%)	0	46 (16.9%)	ID 78 (28.5%)	Q/S 31 (11.4%)	Resp 23 (8.4%)	1.7 (2.3)	36	40
Sudan	6	99 (85–139)	0	0	6 (100%)	0	0	Epidem 3 (50%)	Resp 2 (33.3%)	Renal 1 (16.7%)	0.36 (1.6)	0	2
Somalia	0	0	0	0	0	0	0	N/A	N/A	N/A	0	0	0
Syria	3	80^b^	0	0	2 (66.7%)	0	1 (33.3%)	ID 1 (33.3%)	Q/S 1 (33.3%)	Admin 1 (33.3%)	0	0	2
Tunisia	102	101 (6–5872)	6 (5.9%)	1 (0.98%)	90 (88.2%)	3 (2.9%)	2 (1.96%)	ID 60 (58.8%)	Resp 13 (12.7%)	Heme 7 (6.9%)	1.9 (2.9)	19	28
United Arab Emirates	16	117 (18–386)	1 (6.25%)	0	12 (75%)	0	3 (18.8%)	ID 4 (25%)	Q/S 2 (12.5%)	Trauma 2 (12.5%)	1.46 (1.2)	1	13
Yemen	3	129 (25–387)	0	0	3 (100%)	0	0	Q/S 1 (33.3%)	Education 1 (33.3%)	Cardio 1 (33.3%)	0.47 (0.4)	0	1

*IF* impact factor, *H-index* number of *h* articles that received *h* citations

^a^ The major research field of articles was classified as: administration (Admin), burn, cardiovascular (CVD), cardio-pulmonary resuscitation (CPR), education, endocrine/nutrition, epidemiology (Epid), ethics/end of life/palliative, gastrointestinal, hematology (Heme), immunology/transplant, infectious diseases (ID), neuroscience, organ donation (Org Don) patient and family support (P/F), pharmacology, professional development, quality and safety (Q/S), renal, respiratory (Resp), surgery, and trauma

^b^Sample size available for only one of the two observational studies

The types of patient populations evaluated and the most frequent areas of research for each country are listed in Tables 1 and 2. Adult patients were the most commonly studied group (n = 1119; 72.8%), followed by neonates (n = 267; 17.4%). The most common fields of research were infectious diseases (n = 441; 28.7%) and respiratory diseases (n = 148; 9.6%).

The mean IFs of the journals in which the publications from each country appeared are listed in Table 2, with the H-index, and the study design. The overall mean (SD) IF of the journals that published articles on critical care from the EMR countries was 1.02 (0.7); the mean (SD) IF for countries ranged from 0 to 2, and the number of publications in journals with an IF > 3 ranged from 0 to 36. Although Lebanon did not rank highest in terms of the number of publications (Table 1), the mean IF of the journals in which Lebanese studies were published was the highest. Saudi Arabia had the largest number of publications in journals with an IF > 3 and the highest H-index for publications in critical care and intensive care medicine.

The median sample size in the EMR critical care literature was 120, ranging from 4 patients to > 250,000 observations in surveillance studies. Observational studies accounted for more than two thirds of the publications (n = 1207, 78.5%). Although randomized controlled trials were not common, they comprised one-fifth of the publications from Iran. Meta-analyses were the least common type of study from all countries, with a total of six over the past decade.

### Discussion

Critical care research productivity varied significantly among the EMR countries, with the highest number of publications being from Iran, followed by Saudi Arabia and then Egypt. . However, when assessing research productivity, it is important to adjust for country-related factors such as the population and the state of economic well-being and growth. Previous studies of publications in other biomedical fields in Arab countries ranked Saudi Arabia and Egypt first in terms of absolute number of publications but both were ranked after Iran when including the three countries in the analysis [9–11, 19].

In this study, we evaluated the type of critical care research published from countries in the EMR. Regardless of the economic well-being and population size of the EMR country, the majority of the published studies were observational, with relatively small sample sizes, and primarily evaluated adults. Furthermore, the majority of the research publications were in journals with IF ≤ 3. The research fields of the publications were very diverse but the most common among the majority of the countries were infectious and respiratory diseases. The reason behind having infectious and respiratory diseases as the most common is not clear but this may be reflective of some of the major concerns in critical care in developing countries.

In a study of worldwide research productivity in critical care medicine over a 9-year period (1995–2003), Michalopoulas et al. [14] identified original articles and reviews published in 14 journals directly related to critical care medicine and indexed in PubMed . The authors reviewed a limited number of journals and did not report the research productivity for individual countries. Nevertheless, they also observed an increase in overall research productivity over the years. In addition, they reported significant variation in research output by region, ranging from a total of 161 publications from the African region to 9076 from Western Europe and 8554 from USA.

A similar study was conducted to evaluate national productivity in 20 highly cited journals in critical care (2006–2010) [15]. Studies from high-income countries represented nearly 90% of all articles, with North America being the most productive region. After adjustment for population size, Australia and European countries were more productive in research.

Although many of the critical illnesses encountered in developing countries are similar to those in developed countries, the pathogenesis, management, and outcomes may be different. Studies of critical care in low to middle-income countries indicate inadequate numbers of specialty-trained staff, lack of standardized processes of care, and difficult access to therapies [13, 20]. Research plays an essential role in providing the foundation for evidence-based practice and decision-making at institutional and governmental levels.

The reasons for low research output and low quality of publications in developing countries have been suggested. Constant political turmoil in a region, lack of funding, a “brain drain”, and difficulty in publishing research of local interest in high-IF journals have been described as major barriers [13, 19]. Furthermore, lack of the skills necessary to conduct research and lack of mentorship contribute to poor-quality research that has little chance of being published [13, 19]. In a study evaluating pediatric critical care research in low-and middle-income countries, the main challenges to conducting research were lack of funding, high clinical workload, and limited research support staff [21]. The solutions proposed included increasing research funding, better access to mentors, research training and networks, and improved data collection and medical record systems.

To our knowledge, this is the first comprehensive assessment of critical care research in the EMR. The findings provide a general understanding of the state of critical care research in countries in the Region and guidance for identifying research priorities and needs.

## Limitations

- The search was conducted utilizing PubMed, while other databases such as Embase, HINARI, and Scopus, were not searched.
- Certain articles from the EMR may not have been retrieved from the PubMed search if the name of the country was not mentioned in the abstract.
- The H-index from the Scopus website is based on the publications identified by Scopus and not those that are included in this study and thus there may be some variability.
- The study did not include in-depth content analysis which is necessary to help in setting specific research priorities.

## Abbreviations

WHO: World Health Organization
EMR: Eastern Mediterranean Region
ICU: intensive care unit
IF: impact factor
